# Synthesis, Bioactivity Evaluation and Application of Plant-Based Nanoparticles

**DOI:** 10.3390/molecules28124783

**Published:** 2023-06-15

**Authors:** Govindasamy Rajakumar, Parappurath Narayanan Sudha, Muthu Thiruvengadam

**Affiliations:** 1 Department of Orthodontics, Saveetha Dental College and Hospital, Saveetha Institute of Medical and Technical Sciences, Saveetha University, Chennai 600077, India; 2 DKM College for Women (Autonomous), Vellore 632001, India; 3 Department of Applied Bioscience, College of Life and Environmental Sciences, Konkuk University, Seoul 05029, Republic of Korea

Environmental and biomedical fields have various potential applications for the green synthesis of nanoparticles [1]. The primary objective of green synthesis is to reduce the use of toxic chemicals. In general, the use of biological materials is safe. Furthermore, plants contain reducing and capping agents. In general, the chemical approaches employed are too expensive and involve the use of hazardous and toxic chemicals, which pose numerous environmental risks [1]. For biomedical applications, the biosynthetic route is a safe, biocompatible, and environmentally favourable method for synthesising nanomaterials from plants [1]. This synthesis can be performed using plant elements including leaves, fruits, roots, stems, and seeds. The unique properties of green-synthesised nanomaterials make them excellent candidates for medical applications such as drug delivery, imaging, MRI, etc., with the aim of treating a wide range of diseases [2]. The aim of this Special Issue was to collect papers on the green synthesis (especially plant-based) of nanomaterials, their characterization, and their applications in medicine.

This Special Issue begins with an article by Satti et al. [3], who created biogenic titanium dioxide nanoparticles (TiO_2_ NPs) to control the growth of the pathogenic fungus *Puccinia striiformis* f. sp. tritici (Pst). Biochemical parameters were utilised to evaluate the wheat plants’ response to Pst stress. A proteome analysis devoid of gels and labels was performed to compare protein expressions in pathogen-free (control), pathogen-affected, and TiO_2_-NPs-treated wheat plants infected with Pst. Using bioinformatics, the functional categorization of proteins was determined [3].

Saad et al. [4] reported that shrimp waste could be used to produce chitosan nanoparticles as a source for an environmentally benign nano-nitrogen fertiliser. In addition, they sought to assess the yield potential and its constituents for two wheat cultivars, Misr-1 and Gemeiza-11, using a combination of chitosan nanoparticles (Nan-N) and mineral nitrogen (M-N) fertilisation levels [4]. The evaluated cultivars differed significantly in terms of the majority of growth, yield, and quality variables. Misr-1 had the highest levels of total chlorophyll, spike length, 100-grain weight, grain yield kg/ha, nitrogen, and potassium [4].

Green synthesis can be used for stabilising and reducing cobalt oxide nanoparticles (Co_3_O_4_ NPs) in *Psidium guajava* leaf extracts [5]. Fourier-transform infrared spectroscopy (FTIR analysis), X-ray diffraction analysis (XRD analysis), scanning electron microscopy (SEM analysis), and energy dispersive spectroscopy (EDAX analysis) were used to further characterise the synthesised nanoparticles [5]. In addition, the primary objective of this study was to examine the antibacterial and photocatalytic activity and efficacy of green-synthesised *P. guajava* Co_3_O_4_ nanoparticles on MCF-7 and HCT 116 cells [5].

The next study was carried out on the surface functionalization of phosphorus-rich mineral apatite nanoparticles (ANPs) using thiourea as a source of nitrogen (TU-ANPs) and a co-precipitation technique to increase *Zea mays*’ tolerance to osmotic stress [6]. The resultant thiourea-capped apatite nanostructure (TU-ANP) was characterised using complementary analytical techniques, including EDX, SEM, XRD, and IR spectroscopy [6]. They utilised urea as both a source of nitrogen and a buffer against osmotic stress induced by NaCl in *Zea mays* via germination and seedling foliar application [6].

In another investigation, leaf and fruit aqueous extracts from *Plinia cauliflora* and *Punica granatum* were used to synthesise silver and gold nanoparticles [7]. UV-Vis, TEM, Zeta-potential, and FTIR [7] were used to characterise the nanoparticles. In vitro evaluations of the antimicrobial activity of synthesised nanoparticles were also conducted [7].

Ajlouni et al. [8] described the synthesis of silver nanoparticles (AP-AgNPs) with *Anthemis pseudocotula* Boiss. Various characterization techniques, including XRD, UV-Vis, FT-IR, TEM, SEM, and EDX, were employed to characterise the synthesised AP-AgNPs [8]. In addition, the antimicrobial and anti-biofilm properties of the as-prepared green synthesised AP-AgNPs were evaluated using selected bacterial and fungal strains during this investigation [8].

*Morinda officinalis* polysaccharides (MOP) were used as a dispersant to create selenium nanoparticles (Se-MOP) in the final study [9]. The zeta potential was measured to determine the stability, and UV and ATR-FTIR were employed to determine the binding types of selenium and MOP. TEM was used to observe the morphology. In addition, the inhibitory effect on five selected cancer cells (HepG2, MCF-7, AGS, PC9, and HCT8) was evaluated, revealing that all five cancer cells were inhibited in a remarkable manner [9]. The mechanism may be that Se-MOP inhibits cancer cell proliferation by arresting the cell cycle in the G0/G1 phase [9]. The results revealed that the solitary stimulation of Se-MOP and synergistic stimulation with PHA or LPS increased the immune capacity and enhanced immunity by increasing the expression of cytokines [9].

In summary, this Special Issue provides the most recent developments in nanoparticle research for biomedical applications. We hope that readers will find these articles informative, and useful for their research.
